# Protein and DNA-based assays as complementary tools for fish allergen detection* 

**DOI:** 10.5414/ALX01485E

**Published:** 2017-08-04

**Authors:** A. Kuehn, C. Hilger, T. Graf, F. Hentges

**Affiliations:** 1 Laboratory of Immunogenetics and Allergology, CRP-Santé, Luxembourg; 2 Unité d’Immunologie-Allergologie, Centre Hospitalier de Luxembourg, Luxembourg

**Keywords:** Allergen detection, DNA, ELISA, fish allergy, parvalbumin, PCR, protein

## Abstract

Background: Fish is one of the most important, allergenic foods worldwide. Parvalbumin is the well characterized, major allergen in fish muscle. In this study, we developed a protein- and a DNA-based method for the sensitive detection and authentication of eight commonly consumed fishes in food and compared their applicability. Methods: Fish parvalbumins were purified. Polyclonal, anti-parvalbumin antibodies were raised in rabbits and mice. Protein extracts from food were analyzed by quantitative ELISA. Parvalbumin genes were cloned and sequenced for the design of parvalbumin gene-specific PCR-primers. DNA extracted from food was subjected to specific PCR. Results: Increasing parvalbumin contents were quantified by ELISA in fresh fish, in the order of tuna < mackerel < cod < salmon/trout < redfish < carp < herring. The parvalbumin content of processed fish was up to 67% lower than in fresh fish. In spiked food samples, 1 to 15 ppm fresh fish and 30 to 170 ppm processed fish were still detectable by ELISA. The eight fishes were identified by specific PCR using 0.2 to 10 ng fish DNA. PCRs detected still 3 ppm fresh fish and 30 to 150 ppm processed fish in spiked samples. Conclusions: Both the protein- and the DNA-based method have sufficient sensitivity to protect fish-allergic consumers. The ELISA allows allergen quantification, while the PCR identifies the fish present in the food. The detection limits of both methods vary depending on different factors. Both methods need to be carefully validated for each fish and fish product when used in detection assays.

*Based on a lecture given on the occasion of the 6^th^ German Allergy Congress, Wiesbaden, 2011.

German version published in Allergologie, Vol. 35, No. 7/2012, pp. 343-350

## Introduction 

Fish allergy is one of the most important IgE-mediated food allergies worldwide [23]. The prevalence of fish allergy depends on regional eating habits. Studies suggest that up to 3% of the general population are affected [21]. Clinical symptoms vary strongly between individual patients: from mild reactions to life-threatening anaphylaxis [2, 10]. Parvalbumin is the major allergen in fish muscle [27]. Most patients with fish allergy have specific IgE antibodies against parvalbumin [29]. Parvalbumins are small, water-soluble, extremely stable proteins. The content of parvalbumin in fish muscle depends on the species and is highly variable [16]. Most patients with fish allergy react to various species of fish [29]. This cross-reaction has been explained by conserved IgE epitopes of the parvalbumin molecule [9]. Cases of monosensitivity have also been reported [7, 14]. These species-specific allergies were triggered by allergens other than parvalbumin. In the meantime, it has been shown that clinical monosensitivity can also be explained by IgE antibodies against single fish parvalbumins [17]. 

So far, no specific immunotherapy is available for patients with fish allergy. The strict avoidance of fish products is the only remedy at the moment. However, unknown eating of fish components can still cause allergic reactions [1]. In the European Union, the specific labeling of fish and fish products is compulsory [28]. Any (possible) amount of fish has to be disclosed on the product label [6]. The manufacturer is also obligated to indicate the species of fish, the geographic origin, and the method of production [5, 20]. This specific labeling protects the consumers’ interests. 

Reliable analytical assays are necessary to warrant the adherence to this labeling obligation in two ways: the detection of fish in general and the detection of the specific fish species. Both protein-based and deoxyribonucleic acid (DNA)-based assays are suited for analysis [15]. 

Protein-based assays allow for the simultaneous quantification of fish proteins. The enzyme linked immunosorbent assay (ELISA) is based on the use of specific antibodies. Fish parvalbumins have already been demonstrated and quantified using ELISA [3, 8, 16], but for the identification of the fish species, ELISA is not really suitable. Other protein-analytical assays have been described instead [18, 20]. 

DNA-based assays are often used in food analytic tests. DNA is suitable as a target molecule as it is present in each cell and very stable [15]. Even small numbers of DNA copies can be detected using specific polymerase chain reaction (PCR). For the detection of potentially allergenic fish in food, various PCR assays have been described [4, 11, 25]. DNA-based assays for the identification of fish species using direct or real-time PCR are the most commonly used assays in this context [4, 12]. 

The DNA- or protein-based assays described in the literature have only been validated for certain species of fish or for only few food samples. Our study aimed at developing a protein- and DNA-based assay for the sensitive detection of frequently consumed fish (salmon, trout, carp, cod, mackerel, redfish, herring, tuna) and to compare these two procedures with regard to their use in food samples. 

## Material and methods 

**Samples.** Fish (fresh or processed) and fish-free products (chicken, beef, pork, vegetable stock) were bought in specialized shops in our region. 

**Protein extraction.** Protein extracts were prepared as described elsewhere [16]. All extracts were subject to a buffer exchange in 0.5 mM CaCl_2_, phosphate-buffered saline solution with a pH of 7.2. 

**Fish parvalbumin, anti-parvalbumin antibodies.** Parvalbumins were purified from fish muscle (salmon, trout, carp, cod, mackerel, redfish, herring, tuna) as described elsewhere [16]. Polyclonal anti-parvalbumin rabbit antibodies were produced against a mixture of salmon/cod parvalbumin (polyclonal antibody (PAb) no. 1396) and herring/mackerel parvalbumin (PAb no. 1398). Rabbits were immunized with 200 µg parvalbumin in Freund’s complete adjuvant (Sigma, St. Louis, Mo), followed by three immunizations with 200 µg parvalbumin in Freund’s incomplete adjuvant (Sigma). The IgG antibody fraction was purified using chromatography on A Sepharose from rabbit blood. Polyclonal anti-parvalbumin mouse antibodies were produced in a previous study [16]. A mixture of anti-carp, anti-salmon, and anti-redfish parvalbumin antiserum was used for the detection of fish parvalbumin. 

**ELISA for the detection of fish parvalbumin.** ELISA was carried out using 96-well plates (Nunc, Uden, the Netherlands). 300 ng polyclonal anti-parvalbumin rabbit antibody (PAb no. 1396, PAb 1398) were bound per ELISA plate cavity. The ELISA plate was washed with 0.5% tween/phosphate-buffered saline solution (TBST) pH 7.2 after each step of incubation. Free protein binding sites were saturated with 3% bovine serum albumin (BSA; Sigma) in TBST buffer. Purified carp parvalbumin (2 – 600 ng/mL), protein extracts, or a food matrix spiked with fish extract were incubated overnight (4°C) on the ELISA plate. Polyclonal anti-parvalbumin mouse antibodies (1 : 10,000) were used for detection. Bound mouse antibodies were detected using a horseradish peroxidase-labeled anti-mouse IgG antibody (Sigma). For the color reaction (optical density, 405 nm) we used 2,2’-Azino-bis-(3-ethylbenzothiazoline)-6-sulfonic acid as a substrate. 

**DNA extraction.** Fish DNA was isolated using Genomic Tip 100/G (Qiagen, Hilden, Germany). As a neutral food matrix, vegetarian soup was mixed with 3 – 100 ppm fish (definition: 1 ppm = 1 mg/kg food). For the DNA extraction of food samples, 700 mg of the sample were incubated with 750 µl extraction buffer (0.8% (w/v) sarkosyl, 823 mM NaCl, 23 mM EDTA, 125 mM tris-HCl pH 7.5) and 40 µl proteinase K (56 °C, 3 hours). The extract was extracted with one volume of a chloroform isoamyl alcohol mixture (24 : 1). The DNA was precipitated from the aqueous phase using isopropanol (–20 °C, 6 hours). The precipitated DNA was washed with ethanol and dissolved in sterile water. The purity of the DNA was determined using absorption measurement (A_260/280_). 

**Parvalbumin genes and parvalbumin gen-specific primers.** Parvalbumin cDNA was cloned using rapid amplification of cDNA-ends (RACE) PCR (Clontech, Saint-Germain-en-Laye, France). Parvalbumin genes were amplified using direct PCR (Advantage Polymerase, Clontech). DNA sequencing was carried out (GE Healthcare, Diegem, Belgium) after cloning in plasmid pCR2.1-TOPO (Invitrogen, Groningen, the Netherlands). Gene structures and specific PCR primers were deduced from comparison of the sequence of the parvalbumin cDNA and the parvalbumin genes (AlignIR, LI-COR Biosciences, Cambridge, UK). 

**PCR for the detection of fish parvalbumin.** A 12.5 µL premix of 400 nM primer, 250 µM nukleoside triphosphate (dNTP; Roche, Basel, Switzerland), 3 MM MgCl_2_, and 2.5 µL reaction buffer (Takara, Saint-Germain-en-Laye, France) per cavity was filled in 96-well plates (–20 °C) (Bilatec, Rudolstadt, Germany). If necessary, 0.1 – 300 ng DNA and 0.5 U Taq polymerase (Takara) was added to reach the final volume of 25 µL. DNA amplification was carried out in duplicate in a thermocyler (1 cycle of 2 minutes at 94 °C, 30 – 40 cycles of 30 seconds at 94 °C, 30 seconds at 61 °C, and 1 minute at 72 °C). PCR products were analyzed in a 4% agarose gel (Cambrex, Rockland, ME, USA). 

## Results 

### Protein-based assay: Detection and quantification of fish parvalbumin

In the immunoblot assay, polyclonal rabbit antibodies against fish parvalbumin were able to specifically detect fish parvalbumins in extracts of fresh and processed salmon, trout, carp, cod, mackerel, redfish, herring, and tuna (data not shown). The quantitative ELISA used polyclonal rabbit antibodies as capture antibodies and polyclonal mouse antibodies as detection antibodies for fish parvalbumin. Calibration curves were generated using the purified parvalbumins of the eight fish species within a range of 2 – 600 ng protein/mL. The detection limit for the purified parvalbumins of the eight fish species was 1 ng protein/mL. Parvalbumin was determined in 35 samples of fresh fish, 15 samples of processed fish, and in 10 samples of convenience products (Table 1). The parvalbumin content in fresh fish was on average: 4.5 mg/mL for herring, 3.8 mg/mL for carp, 2.5 mg/mL for redfish, 2.3 mg/mL for salmon/trout, 2.0 mg/mL for cod, 0.6 mg/mL for mackerel, and < 0.05 mg/mL for tuna. The parvalbumin content in cooked fish was 10 – 26 % lower and in smoked/dried fish it was 35 – 67% lower than in fresh fish. In half of the convenience products the amount of fish parvalbumins was < 0.02 – 0.69 mg/g food. In five commercial fish products and in fish-free samples (chicken, beef, pork, vegetable stock) no parvalbumin was detected. 

The detection limit for parvalbumin was determined in a food matrix spiked with 35 extracts of fresh fish and 15 extracts of processed fish (Table 2). With variations according to fish species, 0.1 – 1.5 mg fresh fish/100 g food matrix (1 – 15 ppm) and 3 – 17 mg processed fish/100 g food matrix (30 – 170 ppm) were detectable. In three samples (pickled mackerel, pickled herring, tinned tuna), no parvalbumin could be detected. 

### DNA-based assay: Detection and identification of potentially allergenic fish

**Figure 1.**
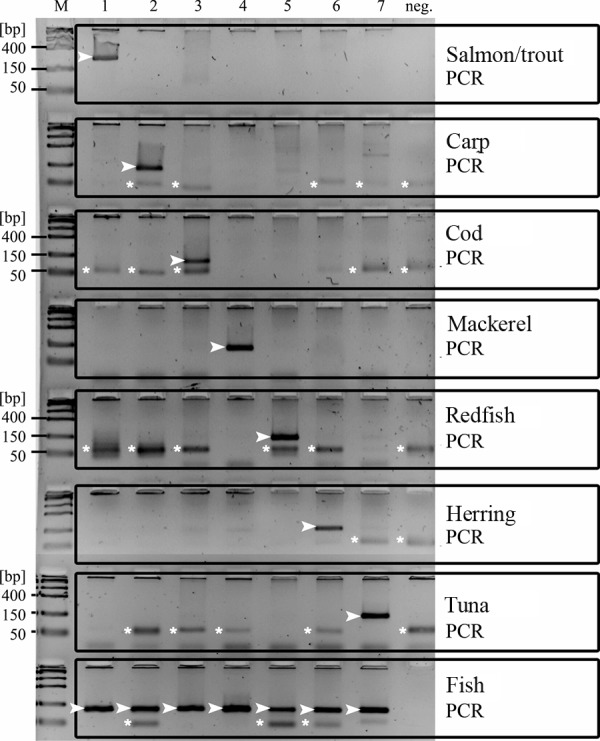
DNA-based assay: Identification and detection of fish. DNA samples in columns 1 – 7 (1: salmon; 2: carp; 3: cod; 4: mackerel; 5: redfish; 6: herring; 7: tuna). bp = base pairs; ► = specific PCR product; *background (dNTP, primer).

Sequences of the cloned parvalbumin cDNA and parvalbumin genes were stored in the EMBL database. For the identification of fish using PCR we used eight primer pairs deduced from exon 1 and intron 1 of the parvalbumin gene in question. For the detection of fish we used primers deduced from exon 2. The identification of fish in the PCR was carried out using DNA from fresh samples (Figure 1). Positive samples identified the fish by presence of a specific PCR product (120 – 220 base pairs) in the reaction mixture. From a series of DNA solutions, 0.2 ng of mackerel DNA, 1 ng of salmon or trout DNA, 2 ng of carp DNA, 3 ng of herring DNA, 5 ng of cod DNA, 5 ng of tuna DNA, and 10 ng of redfish DNA were still detectable in these PCR mixtures. Cross-testing with genomic fish DNA confirmed the specificity of the reaction. The PCR used for the detection of fish (“fish PCR”) amplified a PCR product (240 base pairs) in all mixtures. Cross-testing with genomic DNA from chicken, beef, and pork were negative (data not shown).The specificity of PCR products was confirmed using cloning, sequencing, and comparison with the corresponding parvalbumin gene sequences. 

The detection limit of the PCR for the detection of fish was determined in a food matrix spiked with 35 samples from fresh fish and 15 samples from processed fish (Table 2). Fish DNA was detectable in samples with 0.3 mg fresh fish/100 g food matrix (3 ppm), 0.4 – 0.8 mg cooked fish/100 g food matrix (4 – 8 ppm), and 3 mg smoked or dried fish/100 g food matrix (30 ppm). The detection limit for pickled herring and tinned tuna was > 15 mg fish/100 g food matrix (> 150 ppm). 

## Discussion 

The worldwide yearly per-capita consumption of fish is increasing, and with an increase of 15.7 kg Germany is not an exception (www.fischinfo.de). A high share of fish is consumed in the form of processed fish products. Thus, correct labeling has gained importance [28]. Allergic reactions can be prevented if allergy patients are able to avoid certain products thanks to the specific labeling. However, fish can also be present in other products due to cross-contamination during the manufacturing process. Furthermore, the substitution of high-price by lower-quality fish species is also a problem [19]. Thus, the development of reliable assays for the specific detection of fish and the fish species is of great value for the food industry. 

In our study, we developed an ELISA-design protein-based assay using specific antibodies for the detection of fish parvalbumin. Despite the high homology of fish parvalbumins, not all epitopes are similarly detectable by poly-specific antibodies [8, 16]. Consequently, the sensitivity to detect parvalbumin varied significantly between different species of fish. In this study, we developed polyclonal antibodies that were able to detect the parvalbumins of eight frequently consumed species of fish (salmon, trout, carp, cod, mackerel, redfish, herring, tuna) with a comparable sensitivity. Carp parvalbumin, for which a high cross-reactivity had been reported [26], was used as a standard protein. ELISA was used to confirm the previously determined [16] parvalbumin content of these eight species of fish. In processed fish, the parvalbumin content was found to be 10 – 67% lower than in fresh fish. Parvalbumin was detected in half of the samples derived from convenience products. In highly processed products like pickled or tinned fish, no fish allergen could be detected. Parvalbumins have a high protein stability. Nevertheless, parvalbumin epitopes can be altered or destroyed during the processing of food so that the allergen is not detectable by antibody-based tests [16, 24]. The detection limit of ELISA was 1 – 15 ppm for fresh fish and 30 – 170 ppm for processed fish. According to the allergy-triggering doses described in the literature, a sensitivity in a low ppm range suffices to protect allergic consumers [10, 22]. 

In our study, we developed a DNA-based assay. This PCR method allows for the identification and detection of allergenic fish in food. The analysis of parvalbumin genes of the eight species of fish was essential for the design of specific PCR primers. Although DNA is a stable template, degradations (< 600 bp) have been described under certain circumstances [20]. Primer pairs were chosen to render PCR products of 120 – 240 bp. The identification of the target species salmon, trout, carp, cod, mackerel, redfish, herring, and tuna was carried out with a high sensitivity. The multi-well design allowed for the simultaneous identification and detection of these eight species in a high number of samples. Procedures with a high sample capacity have been described, but they all require much equipment [13, 18]. The detection limit of the DNA-based assay varied and got worse the more a product was processed. This illustrates the degradation of DNA, particularly in pickled products, as described for fish DNA [11]. 3 ppm of fresh fish and 30 – > 150 ppm of processed fish were detectable in the food matrix. The DNA determination in the low ppm range met the demands for use in the detection of potentially allergenic fish. 

The comparison between protein-based and DNA-based assays shows that the sensitivity within a comparable ppm range is adequate to protect consumers who are allergic against fish. The protein-based assay allows for the quantification of the allergen content, but the detection limit varies significantly according to the degree of processing. The DNA-based assay is based on a more stable template, the DNA, and allows for the simultaneous identification of the fish; however, the allergen itself is not detected. Both methods have to be thoroughly validated with regard to the targeted fish species and product. 

## Conflict of interest 

The authors declare that there are no conflicts of interest. 

**Table 1.** Protein-based assay: parvalbumin content in fish and fish products.

**Food**	**Fish/fish product**	**No. of samples**	**Parvalbumin [mg/g]**
Fresh fish	Salmon	4	2.0 – 2.5
	Trout	4	2.0 – 2.5
	Carp	4	2.5 – 5.0
	Cod	4	1.5 – 2.5
	Mackerel	4	0.3 – 0.8
	Redfish	4	2.0 – 3.0
	Herring	4	3.5 – 5.5
	Tuna	6	< 0.05
Processed fish	Salmon, cooked	1	1.5 – 2.0
	Salmon, smoked	2	0.5 – 1.2
	Trout, cooked	1	1.5 – 2.0
	Trout, smoked	2	1.0 – 1.5
	Carp, cooked	1	1.5 – 4.0
	Cod, cooked	1	1.5 – 2.0
	Cod, dried	1	1.0 – 1.5
	Mackerel, cooked	1	< 0.5
	Mackerel, smoked	2	< 0.2
	Redfish, cooked	1	1.5 – 2.5
	Herring, cooked	1	3.0 – 4.5
	Tuna, cooked	1	< 0.02
Convenience products	Salmon, salad	1	ND
	Salmon, spread	1	< 0.05
	Salmon, baby food	1	< 0.04
	Trout, pickled	1	< 0.58
	Trout, salad	1	< 0.02
	Cod, fish sticks	1	< 0.69
	Mackerel, pickled	1	ND
	Herring, pickled	1	ND
	Herring, tinned	1	ND
	Tuna, tinned	1	ND

ND = not detected.

**Table 2.** Protein-based and DNA-based assay: Limits for the detection of parvalbumin using ELISA or for the detection of fish using PCR in a food matrix.

**Food**	Fish/fish product*	**No. of samples**	**ELISA [ppm]**	**PCR [ppm]**
Fresh fish	Salmon	4	1	3
	Trout	4	1	3
	Carp	4	1	3
	Cod	4	1	3
	Mackerel	4	7 – 8	3
	Redfish	4	10 – 15	3
	Herring	4	8 – 10	3
	Tuna	6	2	3
Processed fish, fish products	Salmon, cooked	1	30	5
	Salmon, smoked	2	30 – 50	30
	Trout, pickled	1	130	60
	Trout, smoked	2	30 – 40	30
	Carp, cooked	1	30	6
	Cod, cooked	1	30	4
	Cod, dried	1	30 – 40	30
	Mackerel, pickled	1	ND	50
	Mackerel, smoked	2	60	30
	Redfish, cooked	1	170	8
	Herring, pickled	1	ND	>150
	Tuna, tinned	1	ND	>150

*Used for spiking of the food matrix; ND = not detected; ppm = parts per million (mg/kg food).
